# Evaluation of the detection method by a flotation method using a wire loop for gastrointestinal parasites

**DOI:** 10.1002/vms3.70007

**Published:** 2024-08-29

**Authors:** Aruto Takano, Daikichi Morinaga, Isao Teramoto, Toshimitsu Hatabu, Yasutoshi Kido, Akira Kaneko, Takeshi Hatta, Naotoshi Tsuji, Shigehiko Uni, Kazumi Sasai, Hiromitsu Katoh, Makoto Matsubayashi

**Affiliations:** ^1^ Graduate School of Veterinary Medical Sciences Osaka Metropolitan University Osaka Japan; ^2^ Tri‐Ace Co.,Ltd. Nihonmatsu Fukushima Japan; ^3^ Laboratory of Animal Physiology Graduate School of Environmental Life Natural Science and Technology Okayama University Okayama Japan; ^4^ Departments of Virology and Parasitology Graduate School of Medicine Osaka Metropolitan University Osaka Japan; ^5^ Osaka International Research Center for Infectious Diseases Osaka Metropolitan University Osaka Japan; ^6^ Department of Parasitology and Tropical Medicine Kitasato University School of Medicine Sagamihara Kanagawa Japan; ^7^ Faculty of Science Institute of Biological Sciences Universiti Malaya Kuala Lumpur Malaysia; ^8^ Faculty of Health and Welfare Studies Department of Health Sports and Nutrition Kobe Women's University Hyogo Japan; ^9^ Department of Veterinary Clinical Sciences College of Veterinary Medicine University of the Philippines Los Baños College Laguna Philippines

**Keywords:** egg, flotation method, loop method, oocyst

## Abstract

Infections by gastrointestinal parasites are found in a variety of animals worldwide. For the diagnosis of such infections, the flotation method is commonly used to detect parasitic microorganisms, such as oocysts or eggs, in feces. Instead of adding a flotation solution after the final centrifugation step and using a cover slip to collect the parasites, the method using a wire loop for the recovery of the organisms has been reported as one of alternative methods. However, the recovery rates of microorganisms from the flotation method have not been analysed. In the present study, the utility of a flotation method with the use of a wire loop of 8 mm in diameter (the loop method) was evaluated using different numbers of *E. tenella* oocysts and *Heterakis gallinarum* eggs, and chicken fecal samples collected at the farms. Consequently, we found that the oocysts and eggs in tubes could be collected at a ratio of 2.00 to 3.08. Thus, our results indicate that the loop method is a simple and time saving method, implicating the application for the estimated OPG/ EPG (Oocysts/Eggs per gram) of the samples.

## Graphical abstract

Graphical abstract

► Utility of a flotation method with the use of a wire loop of 8 mm in diameter was evaluated.

► *E. tenella* oocysts and *Heterakis gallinarum* eggs in tubes could be collected at a ratio of 2.00 to 3.08.

► Our results may implicate the application for the estimated OPG/ EPG of the samples as a simple and time saving method.

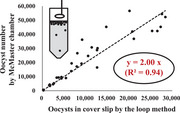

## 1 INTRODUCTION

Gastrointestinal parasites, including protozoans and helminths, are commonly detected in animals and humans around the world. These parasites mainly cause diarrhoea, which can be watery and/or bloody, and they can sometimes also cause death in cases of infections with huge numbers of parasites or infections by highly pathogenic species (Bernard et al., 2021; Geurden et al., 2005; Mesa‐Pineda et al., 2021). In livestock, infections by middle‐ or low‐virulent parasites that do not cause clear clinical symptoms may often still reduce the growth of the animals, especially in neonatal or immature animals; these infections thus have a large economic impact by affecting the productivity of farms (Bandyopadhyay et al., 2010; Kouam & Ngueguim, 2022). Generally, after the asexual and/or sexual proliferation of protozoans or the fertilisation of helminths in the intestines, the robust forms of the organisms such as oocysts, cysts or eggs are shed into the feces as the final stages for the proliferation of next generations.

Although several methods are available for the detection of the final parasitic stages in feces, the flotation technique is commonly used for the diagnosis of infections. The method is characterised by the separation of parasitic organisms (oocysts, cysts and eggs) from fecal debris and the concentration of the organisms by using a flotation solution (e.g., sucrose or saturated sodium chloride solutions), based on differences in specific gravity (s.g.) of approximately 1.20 (Dryden et al., 2005; Pouillevet et al., 2017). A common and basic protocol for the flotation technique is as follows. Fecal samples are diluted with water, and filtrated through a mesh to remove the large debris. After centrifugation, the sediment is mixed with a flotation solution (s.g. 1.2), and the mixture is centrifuged again. Then, flotation solution is added again until a bulging meniscus is formed at the top of the tube, then a glass cover slip is placed on the bulging meniscus. After the parasites have floated up toward the cover slip on the surface of the flotation solution, the cover slip is removed and placed on a glass slide for examination of the parasites.

The flotation procedures often vary between laboratories and studies, as investigators may choose to modify the procedures to simplify them or to make them more complex to fit the purpose of the study (Mekaru et al., 2007; Pouillevet et al., 2017; Pyziel‐Serafin et al., 2022). Instead of adding a flotation solution after the final centrifugation step and using a cover slip to collect the parasites, the method using a wire loop for the recovery of the organisms has been reported in the past as one of alternative methods (Faust et al., 1939). The surveys of the parasites were conducted in a few reports (Bartlett et al., 1978; Dib et al., 2019; McNabb et al., 1985), and however, the efficacy of parasite recovery by the loop method have not been assessed. In the present study, using different numbers of oocysts of *Eimeria tenella* (an apicomplexan protozoan parasite that infects chickens) and eggs of *Heterakis gallinarum* (a nematode parasite that infects chickens) as parasites, we evaluated the recovery numbers of parasitic organisms from the flotation method using sucrose solution and saturated saline solution.

## 2 MATERIALS AND METHODS

### 2.1 Parasites

The *E. tenella* (OPU strain) that was maintained at Osaka Metropolitan University was used in the present study. The oocysts were prepared as reported previously (Matsubayashi et al., 2012). Briefly, chicks (approximately 1 week of age; Takeuchi Furanjo, Nara, Japan) were orally inoculated with matured oocysts, and the oocysts were purified from the feces by the sugar flotation method and sporulated. They were stored at 4°C until they were used in the experiments.

The nematode parasite *H. gallinarum* was also used in our study. The intestines of 20 chickens (ages unknown) from a farm were collected at slaughterhouses in Fukushima Prefecture. Examinations of the contents from each region of the intestine by the direct smear and flotation methods (Ekawasti et al., 2019) detected adult worms and eggs only in the ceca. The detected adult worms were identified as *H. gallinarum* based on the morphology, for example, the body length, spicule structure and caudal alae (Biswas et al., 2021; McDougald, 2020; Yevstafyeva et al., 2018). These eggs were isolated using the same purification method that was used for the *E. tenella* oocysts described above.

### 2.2 Preparation of fecal samples containing parasites

For the evaluation assays in the present study, fecal‐parasite solutions as model samples were prepared as follows. Freshly dropped feces of 14‐week‐old chickens were collected, which were kept at the Poultry Products Quality Control Lab (Fukushima, Japan**)**. They were confirmed to be negative for any parasites by the sugar flotation method (Ekawasti et al., 2019). Ten grams of the pooled feces was diluted in distilled water to a final volume of 90 mL, and filtered through gauze. One millilitre of parasitic (*E. tenella* or *H. gallinarum*) solution was added to 9 mL of the filtrated fecal solution. Fecal‐parasite solutions (10 mL each) were prepared with different concentrations of parasites: 5.0 × 10^2^ to 5.0 × 10^4^ oocysts/mL for *E. tenella* and 3.5 × 10^3^ or 7.0 × 10^3^ eggs/mL for *H. gallinarum*.

### 2.3 Assessment procedures for floating parasites in sugar or saturated saline solution

**FIGURE 1:**
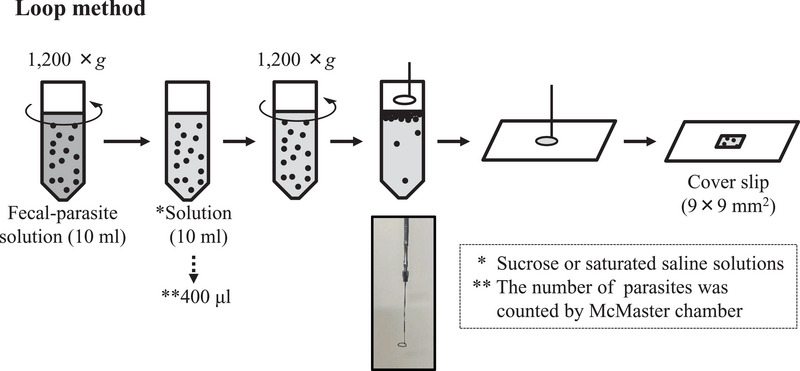
Depiction of the flotation method protocols using sucrose or saturated saline solution. The loop method examined in the present study, which was modified based on a previous report (Faust et al., 1939). Framed picture is the loop used in the present study.

To evaluate the floating numbers of parasites in sucrose solution and saturated saline solution (specific gravity of 1.2), the loop method was conducted as shown in Figure 1. Ten‐millilitre fecal‐parasite solutions containing the oocysts of *E. tenlla* or the eggs of *H. gallinarum* as described above were used. After centrifugation at 1200 × *g* for 5 min, the supernatant was removed, and sucrose or saturated saline solution was added to a final volume of 10 mL and mixed completely. Then, 400 µL of the solution was aliquoted, and the exact numbers of oocysts were determined two times using an McMaster EPG chamber (Fujihira Industry, Tokyo, Japan) under light microscopy at a magnification of ×200 (Nikon, Tokyo, Japan) according to the manufacturer's instructions. After the centrifugation, the upper surface of the tube was carefully collected using a wire loop with a diameter of 8 mm. The surface solution containing the oocysts was transferred from the loop onto a glass slide and covered with a cover glass (9 × 9 mm^2^, Matsunami Glass).

The number of oocysts in the entire field of the cover glass was counted under the microscope for slides containing less than 250 oocysts. For slides containing 250 to 500 oocysts, the number was calculated by counting the number in a 9 to 20‐mm^2^ area. For slides containing more than 500 oocysts, the number was calculated by counting the number in a 4.5 to 5.0‐mm^2^ area. The results were compared to the total number of oocysts in the tubes determined with the McMaster chamber.

### 2.4 Evaluation of fecal samples from chicken farms

A total of 153 dropped fecal samples were collected from 17 broiler farms (ages of the chickens: 13 to 43 days old) located in Miyazaki Prefecture. They were selected as samples positive for *Eimeria* spp. (unknown species). Distilled water was added to 1 to 5 g of the fecal samples to obtain 10‐fold dilutions. The loop method (protocol D) shown in Figure 1 was conducted using 10‐mL dilutions after filtration, and the number of oocysts of *Eimeria* spp. was analysed.

## 3 RESULTS AND DISCUSSIONS

### 3.1 Dose‐dependent recovery numbers by the loop method

**FIGURE 2:**
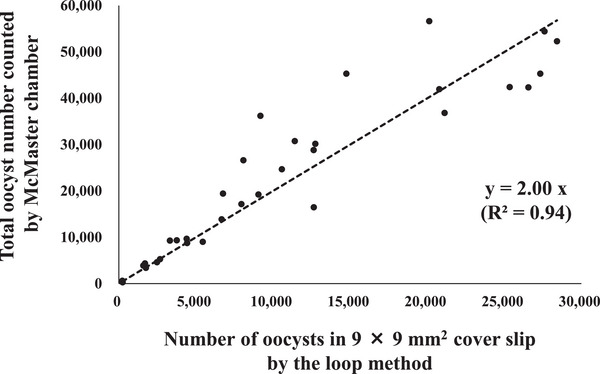
Diagram of the correlation between the oocyst numbers recovered by the loop method and the total numbers counted using a McMaster chamber. The *X* axis shows the number of oocysts in the cover glass (9 × 9 mm^2^) in the loop method. The *Y* axis shows the total number of oocysts in the tubes before the loop was used, as determined with a McMaster chamber.

To evaluate the usefulness of the loop method, the recovery numbers from fecal‐parasite solutions containing a range of *E. tenella* oocyst numbers, from 5.0 × 10^2^ to 5.0 × 10^4^ oocysts, were examined. We found that the total number of oocysts under the 9 × 9‐mm^2^ cover glass positively correlated with the counts from the McMaster chamber (Figure 2). Although slides with a lower number of oocysts (< 500) could not be assessed, because they were not counted with the McMaster chamber, a straight approximation line could be drawn (*y* = 2.00*x*; *R* ^2^ = 0.94). With the use of the saturated saline solution, the solution crystallised rapidly before the entire field could be observed, and the number of oocysts could therefore not be determined. Thus, the further analyses were performed using the sucrose solutions.

**FIGURE 3:**
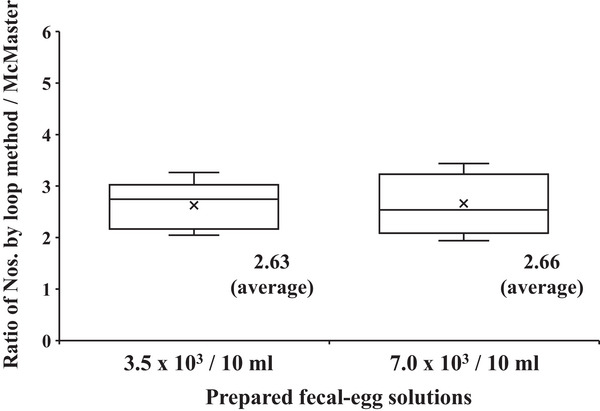
Box‐and‐whisker diagram of the recovery rates of *H. gallinarum* eggs by the loop method. The *Y* axis indicates the ratio of the total number counted with the McMaster chamber to the number of eggs in the 9 × 9‐mm^2^ cover glass after the loop method. The fecal‐egg solutions were prepared at two concentrations, that is, 3.5 × 10^3^/10 mL (left) and 7.0 × 10^3^/10 mL (right). The medians are shown as lines in the boxes, and the averages are indicated by ‘×’.

In the experiments using the fecal‐parasite solutions of *H. gallinarum* eggs at 3.5 × 10^3^ or 7.0 × 10^3^ eggs/10 mL, the ratios of the numbers determined from the McMaster chamber to those from the loop method were 2.63 on average (2.74 as the median) and 2.66 on average (2.53 as the median), respectively (Figure 3). The ratio for *H. gallinarum* eggs was higher than that of *E. tenella* oocysts. Although the reason for this difference could not be determined, the amount or sizes of the debris in the prepared fecal samples might have affected the results. Alternatively, it may have been due to the sizes of the parasites; *E. tenella* measures 20.0 to 26.5 µm in length and 17.0 to 22.0 µm in width (Railliet & Lucet, 1891) while *H. gallinarum* measures 63.6 to 69.3 µm in length and 38.2 to 39.9 µm in width (Yevstafyeva et al., 2018). The eggs of *H. gallinarum* are larger than the oocysts of *E. tenella*, and thus, the debris in the feces may have hindered the flotation of the eggs more than that of the oocysts.

### 3.2 Evaluation of fecal samples from chicken farms

**FIGURE 4:**
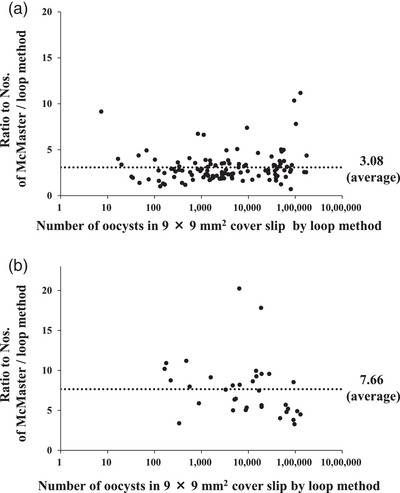
Scatter diagram of the ratios of the total numbers of oocysts counted using the McMaster chamber to those from the loop method in the fecal samples collected at chicken farms. The *X* axes indicate the number of oocysts in the 9 × 9‐mm^2^ cover glass from the loop method. The *Y* axes show the ratio of the total number of oocysts in the tubes as determined using the McMaster chamber to the number from the loop method. Panels A and B show the results of samples without and with mucus, respectively, in the final centrifugation. Dotted lines show the average ratios.

We evaluated the loop method by using fecal samples collected at chicken farms. During the analysis, we found two types of fecal characteristics at the final centrifugation stage in the loop method. Among 153 samples, mucus was clearly found at the top layer of the tubes in 35 samples; the mucus formed a layer thicker than 1 mm in the tubes. Thus, the samples were categorised into those without or with mucus, and analysed separately (Figure 4). In the fecal samples without and with mucus, the ratios of the numbers determined from the McMaster chamber to that from the loop method were 3.08 ± 1.70 and 7.66 ± 3.63 on average, respectively. Thus, fecal samples with mucus could be implicated to reduce the detection by the flotation methods including the loop method. In such cases, mucus‐depleting agents, such as polyethylene glycol, might be useful to remove the mucus.

### 3.3 General discussion

As a diagnostic method for infections by gastrointestinal parasites, the flotation method is conducted worldwide using feces for the detection of microorganisms (oocysts, cysts or eggs), and more recently, flotation solutions, such as sugar and saturated saline solutions, are largely used. To date, many comparative studies of flotation protocols have been reported, mainly through the evaluations of specific gravities, reagents for flotation solutions, and a wide range of protozoans and helminths. Although the reasons remain unknown, the loop method has not been widely used since it was introduced in 1939 (Faust et al.). As far as we know, the method has been used only in a few reports (Bartlett et al., 1978; McNabb et al., 1985), and its sensitivity, including quantitative assessments, has never been analysed. This may be one of the reasons why researchers hesitate to use the loop method.

In the present study, we used the oocysts of *E. tenella* and eggs of *H. gallinarum*. Although we did not evaluate the methods using oocysts or eggs of other parasites, it has been reported that most oocysts or eggs of parasites (nematodes and coccidia) could be floated in solutions with a specific gravity of 1.22 (O'Grady & Slocombe, 1980). The loop method is simple, and saving the time, by being composed of largely three steps: (1) dilution of fecal samples, (2) centrifugation using flotation solution, and (3) picking up the parasites using the loop. Unlike the commonly used flotation method, the addition of flotation solutions after the centrifugation is not needed, in order to form meniscuses and to collect naturally floated up organisms by putting a cover glass. Although comparative data among the methods is not available, the loop method may be more sensitive than the common flotation methods by the deletion of these steps.

For counting the exact number of parasitic organisms, such as oocysts and eggs, in feces, chambers like the McMaster chamber have been preferably used. Here, we were able to more easily detect the parasites under a microscope, and we found that the oocysts and eggs in tubes could be collected approximately at a ratio of 2.00 to 3.08, implicating the application for the estimated OPG/EPG (Oocysts/Eggs per gram) of the samples. Our results indicate that the loop method is a simple and cost‐effective method that may be a useful an alternative tool for the flotation methods.

## AUTHOR CONTRIBUTIONS

I. Teramoto, S. Uni, K. Sasai and M. Matsubayashi constructed the protocols, and additionally T. Hatta, N. Tsuji and H. Katoh contributed to improve the methods. A. Takano, D. Morinaga, T. Hatabu, Y. Kido, A. kaneko, H. Kato and M. Matsubayashi prepared for fecal samples of infected hosts and the parasites. A. Takano and D. Morinaga mainly conducted the fecal examinations based on supporting of the other authors. All authors read and approved the final manuscript.

## CONFLICT OF INTEREST STATEMENT

The authors do not have any conflicts of interest to declare.

## ETHICS STATEMENT

Fecal collection was performed in a noninvasive manner. In the experimental infection study, the animals were treated according to protocols approved by the Animal Care and Use Committee, and in accordance with the Animal Experimentation Guidelines of the Osaka Metropolitan University (approval numbers 15003, 21022, and 22073) or Okayama University (approval number OKU‐2021611).

## Data Availability

The authors confirm that the data supporting the findings of this study are available within the article.

## References

[vms370007-bib-0001] Bandyopadhyay, S. , Mandal, S. , Datta, K. K. , Devi, P. , De, S. , Bera, A. K. , & Bhattacharya, D. (2010). Economic analysis of risk of gastrointestinal parasitic infection in cattle in North Eastern States of India. Tropical Animal Health and Production, 42, 1481–1486. 20411327 10.1007/s11250-010-9582-6

[vms370007-bib-0002] Bartlett, M. S. , Harper, K. , Smith, N. , Verbanac, P. , & Smith, J. W. (1978). Comparative evaluation of a modified zinc sulfate flotation technique. Journal of Clinical Microbiology, 7, 524–528. 566767 10.1128/jcm.7.6.524-528.1978 PMC275056

[vms370007-bib-0003] Bernard, S. N. , Njoga, E. O. , Abonyi, F. O. , Nnadi, P. A. , Ozioko, I. E. , & Ugwuoke, C. U. (2021). Epidemiology of gastrointestinal worm infections in pigs reared in Enugu State, Nigeria. Journal of Parasitic Diseases, 45, 912–920. 34789972 10.1007/s12639-021-01377-y PMC8556453

[vms370007-bib-0004] Biswas, P. G. , Ohari, Y. , Mohanta, U. K. , & Itagaki, T. (2021). Development of a multiplex PCR method for discriminating between *Heterakis gallinarum*, *H. beramporia*, and *H. indica* parasites of poultry. Veterinary Parasitology, 295, 109463. 34023591 10.1016/j.vetpar.2021.109463

[vms370007-bib-0005] Dib, L. V. , Palmer, J. P. S. , de Lima, C. , Ramos, R. C. F. , Bastos, O. M. P. , Uchôa, C. M. A. , Amendoeira, M. R. R. , Fonseca, A. B. M. , Bastos, A. , & Barbosa, A. D. S. (2019). Comparison of four parasitological techniques for laboratory diagnosis of eggs from *Spirometra* spp. in wild mammal fecal samples. Acta Parasitologica, 64, 942–949. 31520294 10.2478/s11686-019-00120-1

[vms370007-bib-0006] Dryden, M. W. , Payne, P. A. , Ridley, R. , & Smith, V. (2005). Comparison of common fecal flotation techniques for the recovery of parasite eggs and oocysts. Veterinary Therapeutics, 6, 15–28. 15906267

[vms370007-bib-0007] Ekawasti, F. , Nurcahyo, W. , Wardhana, A. H. , Shibahara, T. , Tokoro, M. , Sasai, K. , & Matsubayashi, M. (2019). Molecular characterization of highly pathogenic Eimeria species among beef cattle on Java Island, Indonesia. Parasitology International, 72, 101927. 31108220 10.1016/j.parint.2019.101927

[vms370007-bib-0008] Faust, E. C. , Sawitz, W. , Tobie, J. , Odom, V. , Peres, C. , & Lincicome, D. R. (1939). Comparative efficiency of various technics for the diagnosis of protozoa and helminths in feces. Journal of Parasitology, 25, 241–262.

[vms370007-bib-0016] Geurden, T. , Claerebout, E. , & Vercruysse, J. (2005). Protozoan infection causes diarrhea in calves. Tijdschr Diergeneeskd, 130, 734–737. 16363207

[vms370007-bib-0009] Kouam, M. K. , & Ngueguim, F. D. (2022). Prevalence, intensity, and risk factors for helminth Infections in pigs in Menoua, Western Highlands of Cameroon, with some data on protozoa. Journal of Parasitology Research, 2022, 9151294. 35592359 10.1155/2022/9151294 PMC9113898

[vms370007-bib-0010] McDougald, L. R. (2020). Internal parasites. In: D.E. Swayne , M. Boulianne , C.M. Logue , L.R. McDougald , V. Nair , & D.L. Suarez (Eds.), Diseases of poultry (pp. 1157–1191). Wiley‐Blackwell.

[vms370007-bib-0012] Matsubayashi, M. , Hatta, T. , Miyoshi, T. , Anisuzzaman, A. M. A. , Yamaji, K. , Shimura, K. , Isobe, T. , & Tsuji, N. (2012). Synchronous development of *Eimeria tenella* in chicken caeca and utility of laser microdissection for purification of single stage schizont RNA. Parasitology, 139, 1553–1561. 22906745 10.1017/S0031182012001072

[vms370007-bib-0013] Mesa‐Pineda, C. , Navarro‐Ruíz, J. L. , López‐Osorio, S. , Chaparro‐Gutiérrez, J. J. , & Gómez‐Osorio, L. M. (2021). Chicken Coccidiosis: From the parasite lifecycle to control of the disease. Frontiers in Veterinary Science, 8, 787653. 34993246 10.3389/fvets.2021.787653 PMC8724208

[vms370007-bib-0014] Mekaru, S. R. , Marks, S. L. , Felley, A. J. , Chouicha, N. , & Kass, P. H. (2007). Comparison of direct immunofluorescence, immunoassays, and fecal flotation for detection of *Cryptosporidium* spp. and *Giardia* spp. in naturally exposed cats in 4 Northern California animal shelters. Journal of Veterinary Internal Medicine, 21, 959–965. 17939549 10.1892/0891-6640(2007)21[959:codiia]2.0.co;2

[vms370007-bib-0015] McNabb, S. J. , Hensel, D. M. , Welch, D. F. , Heijbel, H. , McKee, G. L. , & Istre, G. R. (1985). Comparison of sedimentation and flotation techniques for identification of *Cryptosporidium* sp. oocysts in a large outbreak of human diarrhea. Journal of Clinical Microbiology, 22, 587–589. 2416771 10.1128/jcm.22.4.587-589.1985 PMC268472

[vms370007-bib-0017] O'Grady, M. R. , & Slocombe, J. O. D. (1980). An investigation of variables in a fecal flotation technique. Canadian Journal of Comparative Medicine, 44, 148–154. 7190861 PMC1320050

[vms370007-bib-0018] Pouillevet, H. , Dibakou, S. E. , Ngoubangoye, B. , Poirotte, C. , & Charpentier, M. J. E. (2017). A comparative study of four methods for the detection of Nematode eggs and large protozoan cysts in mandrill faecal Material. Folia Primatologica, 88, 344–357. 10.1159/000480233 29041010

[vms370007-bib-0011] Pyziel‐Serafin, A. M. , Raboszuk, A. , Klich, D. , Orłowska, B. , Sierociuk, D. , & Anusz, K. (2022). Two centrifugal flotation techniques for counting gastrointestinal parasite eggs and oocysts in Alpaca faeces. Journal of Veterinary Research, 66, 389–393. 36349129 10.2478/jvetres-2022-0039 PMC9597935

[vms370007-bib-0019] Railliet, A. , & Lucet, A. (1891). Developpement experimental des coccidies de l'epithelium intestinal du lapin et de la poule. Comptes Rendus Société Biologies, 36, 820–823.

[vms370007-bib-0020] Yevstafyeva, V. A. , Melnychuk, V. V. , Nikiforova, О. V. , Suprunenko, К. V. , Korchan, L. N. , Lokes‐Krupka, Т. Р. , Nehrebetskyi, I. S. , & Korchan, N. І. (2018). Comparative morphology and biology of nematodes of genus *Heterakis* (Nematoda, Heterakidae), parasites of the domestic goose (*Anser anser*) in Ukraine. Regulatory Mechanisms in Biosystems, 9, 229–236.

